# Genome-Scale Metabolic Modeling Elucidates the Role of Proliferative Adaptation in Causing the Warburg Effect

**DOI:** 10.1371/journal.pcbi.1002018

**Published:** 2011-03-10

**Authors:** Tomer Shlomi, Tomer Benyamini, Eyal Gottlieb, Roded Sharan, Eytan Ruppin

**Affiliations:** 1 Computer Science Department, Technion - Israel Institute of Technology, Haifa, Israel; 2 The Blavatnik School of Computer Science, Tel Aviv University, Tel Aviv, Israel; 3 The Beatson Institute for Cancer Research, Glasgow, United Kingdom; 4 The Sackler School of Medicine, Tel Aviv University, Tel Aviv, Israel; University of Virginia, United States of America

## Abstract

The Warburg effect - a classical hallmark of cancer metabolism - is a counter-intuitive phenomenon in which rapidly proliferating cancer cells resort to inefficient ATP production via glycolysis leading to lactate secretion, instead of relying primarily on more efficient energy production through mitochondrial oxidative phosphorylation, as most normal cells do. The causes for the Warburg effect have remained a subject of considerable controversy since its discovery over 80 years ago, with several competing hypotheses. Here, utilizing a genome-scale human metabolic network model accounting for stoichiometric and enzyme solvent capacity considerations, we show that the Warburg effect is a direct consequence of the metabolic adaptation of cancer cells to increase biomass production rate. The analysis is shown to accurately capture a three phase metabolic behavior that is observed experimentally during oncogenic progression, as well as a prominent characteristic of cancer cells involving their preference for glutamine uptake over other amino acids.

## Author Summary

Cancer cells, as opposed to normal cells, produce a substantial amount of energy inefficiently via aerobic glycolysis, even in the presence of sufficient oxygen to support mitochondrial respiration. Despite the fact that this phenomenon, called the Warburg effect, has already been discovered back in 1924, its causes remain poorly understood. Here we utilize a genome-scale human metabolic network model and show that by accounting for cellular capacity for metabolic enzymes, the Warburg effect is a direct consequence of cancer cells' adaptation to fast proliferation. We demonstrate that our model accurately captures several metabolic phenotypes observed experimentally during cancer development, as well as the preference of cancer cells to glutamine uptake over other amino acids.

## Introduction

The Warburg effect, a phenomenon discovered by Otto Warburg in 1924, reflects a shift to an inefficient metabolism in cancer cells, in which an increase in the inefficient production of adenosine 5′-triphosphate (ATP) via glycolysis leads to the secretion of non-oxidized carbons in the form of lactate, even in the presence of oxygen (termed *aerobic glycolysis*) [1], [2]. Specifically, aerobic glycolysis allows the production of only 2 ATP molecules per one glucose molecule, whereas oxidative phosphorylation permits the generation of 32 ATP molecules per one molecule of glucose [3]. Nevertheless, the importance of aerobic glycolysis to cancer cells has been experimentally demonstrated [4], [5].

Over the years, several hypotheses were raised regarding the potential cause of the Warburg effect: (i) Defective mitochondrion hypothesis – suggesting that cancer cells have defective mitochondria and hence rely on glycolysis [6], however subsequent research revealed that mitochondrial function is not impaired in most cancer cells [7], [8]. (ii) Hypoxia– suggesting that tumor hypoxia selects for cells dependent on anaerobic metabolism [9], but previous studies have shown that cancer cells already resort to aerobic glycolysis before exposure to hypoxic conditions [10], [11]. (iii) Avoiding ROS-mediated DNA damage – it was suggested that reducing oxidative phosphorylation in proliferating cells due to the Warburg shift reduces ROS and hence protects cells from DNA damage and subsequent apoptosis [12]. (iv) A game theoretical approach suggesting that the Warburg effect occurs as glycolysis provides higher ATP production rate than oxidative phosphorylation [13], [14], [15]. (v) An approach suggesting that a trade-off between the enzyme-synthesis costs and the ATP production yields of the different pathways that catabolize carbon sources may cause the Warburg effect: the high-yield oxidative phosphorylation pathway also has high enzyme costs, leading to a sub-optimal ATP production strategy, as it has lower production rates than glycolysis [16]. (vi) Metabolic adaptation to fast proliferation - it was argued that as opposed to metabolism in differentiated cells that is geared towards efficient ATP production, the aerobic glycolysis observed in cancer cells is adapted to facilitate biomass accumulation and high proliferation. Accordingly, in order to satisfy the requirements of anabolic metabolism in addition to the production of ATP, nutrients must be used to generate both the carbon building blocks of macromolecules and the reducing power needed for biosynthesis [17].

Previous computational investigations of the Warburg effect studied the role of either energy or biomass production in causing the Warburg effect, focusing solely on central carbon metabolism. For example, the study of Vander Heiden *et al.* manually computed the metabolic requirements for producing one essential biomass precursor, palmitate (a major constituent of cellular membranes) considering the stoichiometry of a few central metabolic pathways. They found that aerobic glycolysis enables maximal palmitate production yield due to specific reducing power requirements. In another recent study, Vazquez *et al.* employed a schematic model of ATP production in human cells (considering two lumped reactions representing aerobic glycolysis and oxidative phosphorylation), elegantly showing that a switch to aerobic glycolysis should result from cellular maximization of ATP production [18]. Their schematic model accounts not only for the stoichiometry of glycolysis and oxidative phosphyrylation but also for the enzyme-volumetric costs of activating these pathways (the latter bounded by the total cellular solvent capacity, also known as a *macromolecular crowding* constraint [19]). A similar approach was previously employed in the study of over-flow metabolism in *E. coli* [20], [21]. Another interesting theory explaining overflow metabolism was suggested by Molenaar *et al.*, where the production costs of the metabolic enzymes involved were accounted for in a self-replicating model [16].

In this paper, we study the causes of the Warburg effect by accounting for both energy production and anabolism of essential biomass constituents, in a genome-scale stoichiometric network model [22] employing enzyme solvent capacity constraints. The usage of a large-scale metabolic network is essential if one aims to correctly account for the inter-connectivity of pathways that produce the various energy and biomass precursors required for proliferation, rather than examining just single factors in isolation, as has been previously performed in [17], [18]. Towards this goal, we rely on a constraint-based modeling (CBM) framework that serves to analyze the function of metabolic networks by solely relying on simple physical-chemical constraints [23]. CBM has already been successfully used in the past to predict the metabolic state of various microorganisms [24], [25], and recently for studying human cellular metabolism [22]. The potential clinical utility of the human CBM model was previously demonstrated by its ability to identify functionally related sets of reactions that are causally related to hemolytic anemia, and potential drug targets for treating hypercholesterolemia [22], to predict metabolic biomarkers in inborn errors of metabolism [26] and to predict a variety of metabolic behaviors of different human tissues, including the brain, liver, kidney and more [27], [28]. Our analysis shows that while strictly stoichiometric considerations are insufficient for explaining the Warburg effect, the incorporation of enzyme solvent capacity constraints successfully predicts the emergence of the Warburg effect. The analysis is shown to accurately predict an experimentally observed metabolic trajectory occurring during oncogenic progression, as well as the preference of cancer cells for a high rate of glutamine uptake.

## Results

### Metabolic requirements of cellular proliferation subject to enzyme solvent capacity constraints lead to the Warburg effect

We utilized a genome-scale human metabolic network that includes 3,742 reactions [22], adding a pseudo *biomass reaction* that represents the production of a pre-defined set of essential biomass precursors required for cellular proliferation, as conventionally done in Flux Balance Analysis (FBA, [29], see Methods). The biomass precursors include amino-acids, nucleotides, deoxy-nucleotides, ATP, lipids, etc (based on prior knowledge of their relative concentrations; Methods). In our simulations, we assume a minimal growth medium with glucose as a carbon source, as glucose is known to serve as a major fuel in cancer cells (below and in Text S1 we show that qualitatively similar results were obtained when considering also the presence of an additional major nutrient taken by cancer cells, glutamine).

To predict plausible metabolic fluxes in cancer, we first employed a standard FBA method to identify a feasible flux distribution that satisfies stoichiometric mass-balance, while maximizing biomass production yield (see Methods). We found that the predicted flux distribution does not display the prime characteristic of the Warburg effect, *i.e.* lactate secretion (see also Text S1). Interestingly, this finding is in accordance with a previous study showing a conceptually similar failure of FBA to predict the Crabtree effect in yeast, in which glucose is fermented into ethanol under aerobic conditions [30]. Thus, stoichiometric considerations alone are insufficient for explaining the Warburg effect and its relation to the metabolic requirements of highly proliferating cells. Notably, these results stand in difference from those presented by Vander Heiden *et al.* [17], claiming that strictly stoichiometric considerations directly lead to the Warburg effect due to metabolic demands for cellular proliferation.

A strictly stoichiometric analysis, such as the one presented above, implicitly assumes that metabolic flux rates can be tuned to achieve high biomass production yields, without considering constraints imposed by enzyme concentrations and catalytic rates, which are prime determinants of metabolic flux. Specifically, while cells might be free to regulate enzyme concentrations according to metabolic demands [17], the total enzymes' concentration in the proliferating cells is bounded by the cell's solvent capacity, quantifying the maximum amount of macromolecules that can occupy the intracellular space [18]. To account for the functional effects of this additional fundamental constraint, we follow [18], [21] and extend our stoichiometric genome-scale CBM analysis to compute for each enzyme the concentration required to facilitate the predicted flux, utilizing data on known human enzyme catalytic rates (taken from the literature; see Methods). This modeling approach enables the prediction of metabolic flux distributions that maximize the biomass production rate and concomitantly obey the solvent capacity constraints – rather than predicting flux distributions that only maximize the biomass production yield as done in standard FBA.

**Figure 1:**
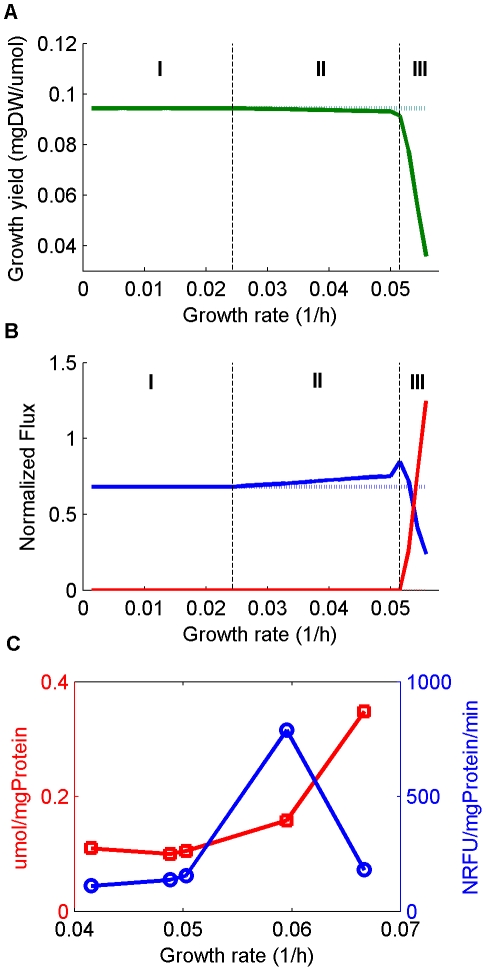
Metabolic behavior across increasing growth rates. (A) Predicted maximalgrowth yield of human cells (per unit of glucose uptake; y-axis) for a range of growth rates (x-axis), based strictly on reactions' stoichiometry (dotted) and by considering also enzyme mass and enzyme solvent capacity (solid). Vertical dashed lines indicate the borders between: phase I (high yield, no lactate secretion), phase II (medium yield, increased oxidative phosphorylation) and phase III (low yield, lactate secretion). (B) Predicted lactate secretion flux (red lines) and oxygen consumption flux (blue lines) for a range of growth rates. Growth rates were manipulated by varying the glucose uptake rate limit from 0 until the uptake value needed to reach the maximal growth rate. Fluxes were normalized by the glucose uptake rate. (C) Experimentally determined lactate secretion rates (red; squares) and oxygen uptake rates (blue; circles) during tumor development of H-RasV12/E1A transformed fibroblasts. NRFU: Normalized relative fluorescence units; see [31] for more details.

**Figure 2:**
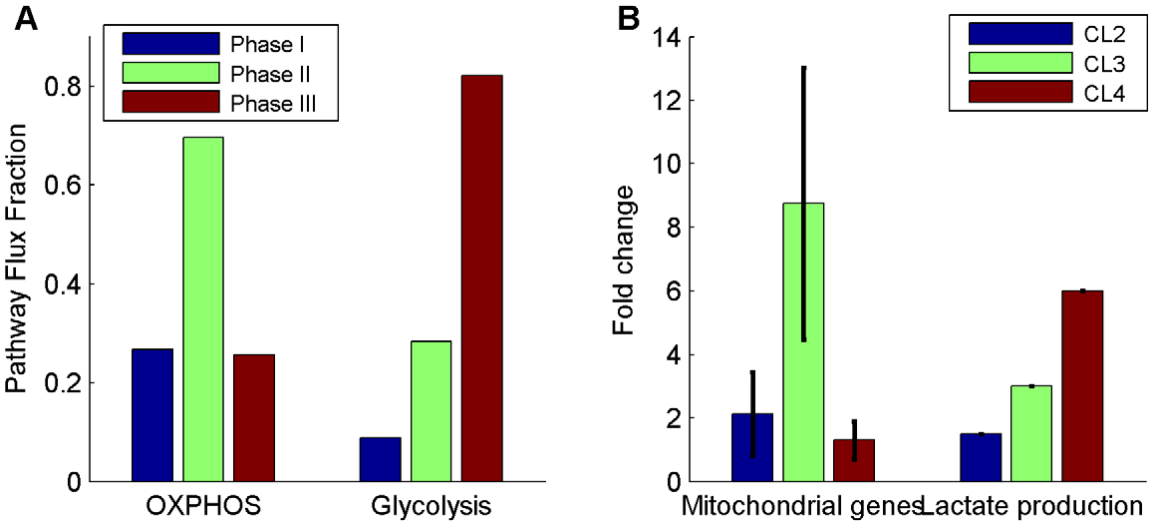
Pathway activity differences. (A) as predicted across phases I-III in the model and (B) based on experimental measurements taken from BJ fibroblast cell lines representing the path towards tumorigenic conversion (CL1-CL4; [32]). The model's predictions are compatible with the experimental evidences for increased glycolytic activity (expressed by increased lactate production) during full cancerous development (phase III, CL4) preceded by an increase in the oxidative phosphorylation (OXPHOS) activity (expressed by the mitochondrial gene expression). Experimental results for CL2-CL4 are given as the fold change relative to the same measurement in the CL1 cell line. In (B), the bars represent the mean fold change for each set of metabolites/genes and the error bars represent the standard deviation.

We applied the approach described above (FBA with solvent capacity constraint) to predict human cellular flux distributions that maximize the biomass production rate. To simulate varying growth rates we performed the optimization across a wide range of different glucose uptake rates. Indeed, under these combined sets of constraints we find that biomass yield does decline at high growth rates – in accordance with the Warburg effect [17]; Figure 1a). Specifically, the predicted metabolic behavior manifests three distinct growth phases (Figure 1b): *(i) optimal yield metabolism* at a growth rate that is below 43% of the maximal possible rate, characterized by low glycolytic vs. high oxidative phosphorylation (OXPHOS) flux (Figure 2a, phase I), with low oxygen uptake rates (Figure 1b, phase I*). (ii) Intermediate yield metabolism* at growth rate between 43-92%, characterized by increased glycolytic and oxidative phosphorylation flux (Figure 1a, phase II), the latter involving a significantly increased oxygen consumption (Figure 1b, phase II). Notably, our prediction for an intermediate phase, involving increased oxygen consumption, presents a remarkable resemblance to two recent experimental studies examining the metabolic activity at different oncogenic progression stages ([31], Figure 1c and [32], Figure 2b). Neither the stoichiometric model [17] nor an analysis using the schematic model of [18] give rise to similar predictions. *(iii) Low yield metabolism* at a growth rate above 92% of the maximal possible growth rate, characterized by a sharp increase in glycolytic flux and a decrease in oxidative phosphorylation (and hence of O_2_ uptake). The increase in aerobic glycolysis flux (Figure 2a, phase III) leads to a rise in lactate secretion rates - a prime characteristic of the Warburg effect (Figure 1b, phase III).

To further validate the plausibility of the model, we examined the correlation between its enzyme concentration predictions (based on predicted flux distributions; see Methods) and mRNA expression values measured for 1,269 metabolic genes across 60 cancer cell lines of the NCI-collection [33]. The enzyme concentrations predicted with FBA accounting for the solvent capacity constraint show significant rank correlations with the gene expression data across the different cancer cell-lines (mean Spearman correlation of 0.28, mean p-value  =  6.5e−21). Notably, the strictly stoichiometric analysis provides significantly lower correlations with the expression measurements (with a mean correlation of 0.1; Wilcoxon p-value  =  3.5e−21), further demonstrating the advantage of the genome-scale approach that accounts for enzyme solvent capacity.

### Explaining the shift to aerobic glycolysis under high proliferation rates

**Figure 3:**
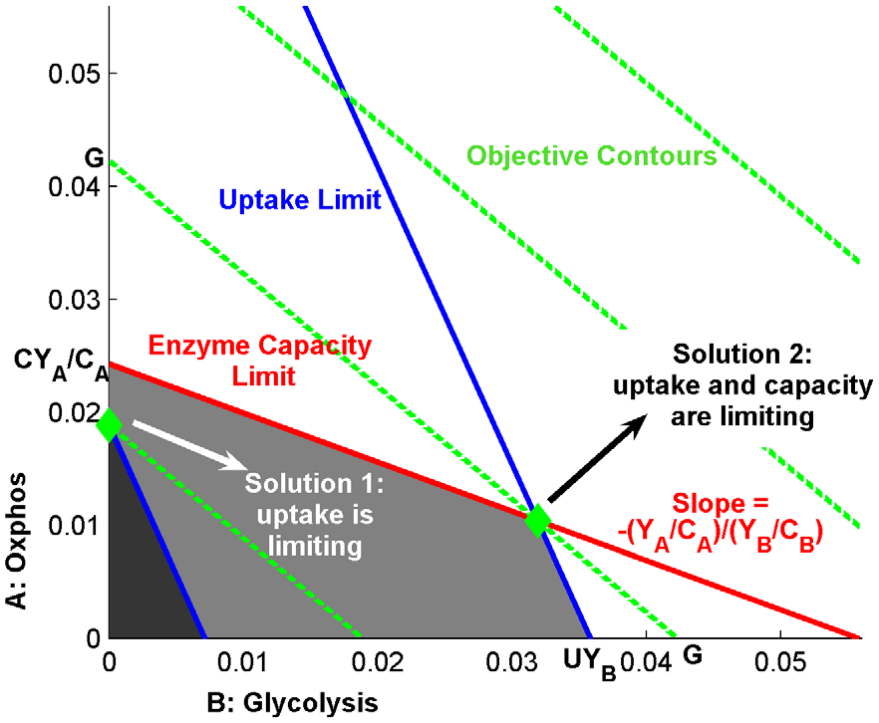
A plane describing the feasible region in our model. The axes (A, B) describe the growth rate obtained from flux distributions A and B, respectively. The blue lines represent two different constraints on the glucose uptake rate, and the red line represents the maximal concentration constraint. Green dashed lines are the contours of the growth rate maximization objective function – the further the line is from the origin, the higher the growth rate. When the glucose uptake U is limiting (dark grey feasible region), the maximal growth rate is obtained via A only (Solution 1; left green diamond). When both the uptake and the enzyme concentration constraints are limiting (light grey feasible region), maximal growth rate (G) is obtained via a combination of A and B (Solution 2; right green diamond), resulting in a shift to a less efficient metabolism and lactate secretion. This can be explained by the fact that the slope of the growth-rate (middle green) line (-1) is larger than the slope of the enzyme concentration limit (red) line (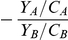), that is the yield-to-cost ratio of flux distribution B is greater than that of flux distribution A (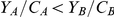).

The shift towards *low yield metabolism* at high growth rates can be intuitively explained considering, on one hand, a flux distribution A with high growth yield (*Y_A_*) and high ‘cost’ in terms of the required enzyme concentrations per unit of glucose uptake (*C_A_*), and, on the other hand, a flux distribution B with a lower growth yield (*Y_B_*) and lower cost (*C_B_*). Considering a bound on the total enzyme concentration cost, one can observe that when the glucose uptake is unlimited flux distribution *B* will provide a higher growth rate if its growth yield normalized by its cost is higher than that of flux distribution A (*i.e.* 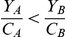; Figure 3). When the glucose uptake rate is limited, maximal growth rate is achieved solely via flux distribution *A* or by a combination of *A* and *B*.

Concretely, analyzing the results of our model, flux distribution *A* stands for a typical metabolic state in phase I, which is characterized by high mitochondrial oxidative phosphorylation, with a high growth yield of 0.094 and a high cost of 0.302 (with a yield to cost ratio of 0.31). Flux distribution B stands for a typical metabolic state at phase III, which involves a high rate of aerobic glycolysis, with a low growth yield of 0.035 and a low cost of 0.050 (yield to cost ratio  =  0.7). These values indeed transcribe to a higher growth yield per unit of concentration cost of the enzymes participating in B, as alluded above. Figure 3 shows that, indeed, at low growth rates the glucose uptake rate is the sole limiting factor and hence the high yield oxidative phosphorylation route is taken; in contrast, at higher growth rates, the enzyme concentration constraint takes effect, and mixed solutions involving lactate secretion are necessarily formed. Notably, the predicted flux distributions across the range of growth rates described in this paper cannot be obtained from linear combinations of just two states (as in the above simplified example), but are rather composed of multiple flux distributions with different growth yields per concentration cost (as evident for example by the non-linear curve showing the predicted oxygen uptake rates across growth rates; Figure 1b). Thus, the flux distributions actually obtained in genome-scale models markedly differ from those that can be captured by a simplified analysis that describes the transition between just two metabolic states with different growth yields as above, or as in a previous study of Vazquez *et al.* [18].

### Metabolic adaptation to fast proliferation leads to a preference to high glutamine uptake rates

**Figure 4:**
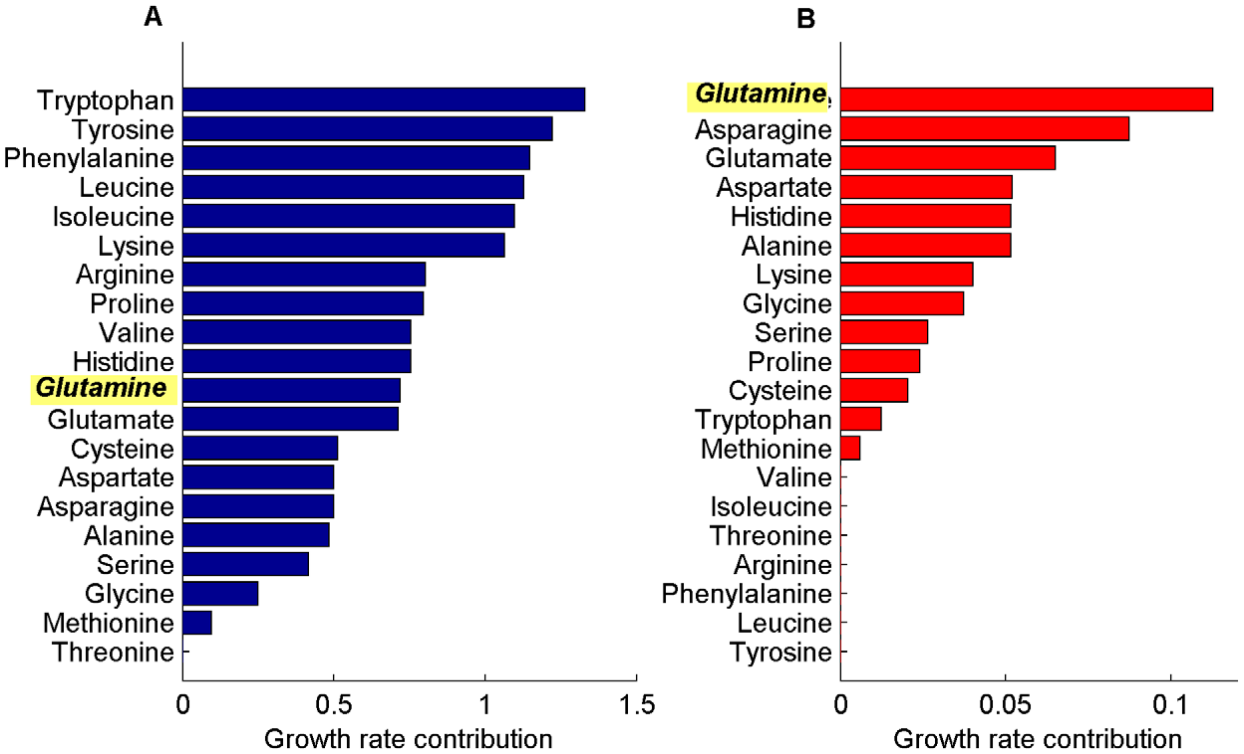
Amino-acid growth rate contribution. The increase in proliferation rate achievable by the increased uptake of each of the 20 amino-acids in addition to glucose (relative to the baseline growth rate achieved when only glucose is available), as predicted by the stoichiometric model (A) and by the model accounting for the solvent capacity constraint (B). Glutamine uptake (highlighted in yellow) enables to achieve the highest increase in growth rate according to the solvent capacity model, in agreement with experimental data showing preference for high glutamine uptake rates in cancer.

The role of glutamine in cancer has been a topic of major interest as cancer cells are known to have a significant high glutamine uptake rate [34]. Repeating the previous analyses in the presence of both glucose and glutamine in the growth media shows qualitatively similar results to those described above. However, as expected, the addition of glutamine yields a higher maximal biomass production rate than the one obtained when only glucose was available in the medium (Text S1). To investigate the preference of cancer cells specifically to glutamine over other amino-acids, we applied FBA analysis to predict the contribution of each amino-acid separately to biomass production in the human model that accounts for enzyme solvent capacity constraints (Methods). We find that, indeed, the contribution of glutamine to the proliferation rate is markedly higher than that of all other amino-acids (Figure 4B). We further show that this result is robust to changing the bound on maximal amino-acid uptake rate, and that it remains valid across a large number of random samplings of enzyme turnover rates (Text S1). Repeating this analysis without accounting for enzyme solvent capacity constraints (*i.e.* by considering only the network stoichiometry in the vanilla FBA model) fails to predict the preference for glutamine (Figure 4A).

We carefully examined the flux distribution obtained with glutamine in the growth medium (achieving a growth rate of 0.062 1/h) vs. the one obtained with glutamate (growth rate  =  0.056 1/h). Interestingly, when glutamate is present in the medium, a large quantity of it is transformed into glutamine in an ATP consuming reaction catalyzed by the enzyme glutamine synthetase (EC 6.3.1.2). This satisfies the glutamine biomass requirement as well as the production of nucleotide precursors, among others. When removing the ATP requirement from this reaction, the growth rate achieved with glutamate in the medium increases to 0.059 1/h, which explains 50% of the growth rate difference. Notably, while this provides some intuitive explanation for the predicted preference for glutamine, we cannot identify a simple explanation for the entire effect due to the high complexity of the network model employed.

## Discussion

Metabolic adaptation to elevated growth requirements during cancer development has been recently suggested as the possible cause of the Warburg effect, a long-standing enigma of cancer metabolism. In this work we rigorously study this hypothesis using a genome-scale human metabolic model and demonstrate that stoichiometric considerations solely are insufficient to explain the shift to inefficient metabolism, in difference from recent claims [17]. However, integrating these constraints in a genome-scale model of human metabolism together with a constraint on enzyme solvent capacity does lead to the emergence of the Warburg effect at high proliferation rates. Furthermore, it accurately predicts a three phase metabolic behavior experimentally observed during oncogenic progression, as well as a marked preference to a high uptake rate of glutamine.

The importance of enzyme solvent capacity in metabolic modeling has already been recognized in the earlier work of Beg *et al.* [20], where applying such a constraint to the *E. coli* model improved phenotypic predictions. In their work, however, Beg *et al.* assumed an upper bound on the total cell-volume occupied by metabolic enzymes, as opposed to the method introduced here where we assume a bound on the enzyme mass per cell mass (*i.e.* a bound on enzyme fractional concentration). In order to account for enzyme volumes, Beg *et al.* estimated enzyme volumes by assuming a uniform *specific volume* parameter (representing the ratio between enzyme mass and volume) for all enzymes. Here, we employed a simpler approach that does not depend on specific volume estimations, and explicitly constrains the total sum of enzyme mass. Notably, we further tested the effect of accounting for volumes instead of masses, and obtained results which are very similar to those obtained with masses only (Text S1).

In a recent study by Molenaar *et al.* [16], a metabolic shift at high growth rates was predicted based on a general self-replicating model. Another recent work (by Vazquez *et al.*) already pointed to the significance of the solvent-capacity constraint in explaining the Warburg effect [18]. Notably though, the work presented here provides a marked contribution over both studies: First, both employ abstract small-scale models. Specifically, the Vazquez *et al.* work introduces a schematic model of ATP production in central metabolism including just a handful of variables. Furthermore, similarly to the work of Pfeiffer *et al.*, their work does not explicitly account for the entire biomass composition and the associated energy requirements. In contrast, here we study a genome-scale biomass producing human model that, despite the scores of alternative biomass and energy production pathways existing in the human network, successfully shows that highly proliferating cells such as cancer cells are forced to display Warburg related phenotypes at high growth rates (phase III). Additionally, and in contrast to the small-scale models, our genome-scale model correctly predicts an experimentally observed transitional phase (II). Furthermore, on a mechanistic level, the genome-scale metabolic description provided by our analysis is significantly correlated with the gene expression patterns across the wide array of NCI-60 cancer cell-lines (much stronger than the association displayed by the stoichiometric model alone), a result which could not have been predicted by the Vazquez *et al.* model. Lastly, the model was able to predict the marked contribution of glutamine to rapid cellular growth. As a further demonstration of the robustness of our results, we repeated the analyses using a model accounting for maintenance ATP production, obtaining qualitatively similar results (Text S1).

While the data on reactions' stoichiometry is considered accurate and comprehensive, enzyme kinetic constant data are noisy and are currently available for only about 15% of the reactions in the model. In the analysis presented here, we addressed this problem by assigning enzymes with missing turnover rates with the median rate computed over the set of known turnover rates. Notably, the model's main findings are robust to random sampling of turnover rates from a distribution of known rates, as shown in Text S1. However, repeating the analysis when assigning all reactions in the model with the median turn-over rate shows no Warburg characteristics - testifying to the importance of utilizing known turnover rates even if this data is sparse. Future measurements of additional enzyme turnover rates and improved methods for accurately predicting these parameters (*e.g.* [35]) are expected to further refine the predictions of cancer metabolic phenotypes using stoichiometric metabolic models with an enzyme solvent capacity constraint.

In our work we accounted for a solvent capacity constraint assuming a limited protein mass per cell, without considering the effect of enzymes' sub-cellular compartmentalization. To investigate how the latter would affect our predictions, we repeated the analysis while considering separate solvent capacity constraints for cytoplasm and mitochondria (Text S1), yielding quantitatively similar results to those described above. The incorporation of solvent capacity constraints for different cellular compartments may lead to further improved prediction accuracy in the future, when additional data on enzyme turnover rates becomes available. Specifically, the addition of membrane-specific constraints may be a promising direction, as many metabolically important proteins are confined to membranes (e.g. those of respiratory chain and membrane biosynthesis).

The presented modeling approach is likely to contribute to more accurate metabolic modeling of highly proliferating human cells in general (as was already shown regarding genome-scale models of microorganisms [21]) and of cancer cells. The latter may be in turn utilized for anti-cancer drug target prediction and specifically, for predicting drugs that work to reverse the Warburg effect. While the current analysis has relied on the available human generic model, future studies may utilize a similar methodology in modeling the metabolism of specific cancers. These may be generated by integrating cancer-signature expression data with the generic human model to carve out different cancer types models (using methods such as those outlined in [27] or [28]), and thus further advance the development of anti-cancer drugs specific to different cancers.

## Materials and Methods

### Modeling biomass production using a stoichiometric model

The Duarte *et al.* [22] human genome-scale metabolic model, accounting for 1,496 ORFs, 3,742 reactions and 2,766 metabolites, was used. The metabolic network is represented in a 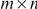 stoichiometric matrix *S*, where *m* is the number of metabolites, *n* is the number of reactions, and 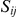 represents the stoichiometric coefficient of metabolite *i* in reaction *j*. Biomass production was modeled by adding a new growth reaction to the human model: this reaction was compiled using the steady state concentrations of 30 biomass compounds including amino acids (0.78 g/gDW; [36], [37]), nucleotides (0.06 g/gDW; [38]), lipids (0.16 g/gDW; [39]) as well as the growth-associated energy requirement (24 mmol/gDW of ATP; [40]). Essential amino acids were not accounted for since they were assumed not to take active part in the metabolic model besides flowing directly into the biomass reaction. The full list of biomass metabolites and their relative concentrations is available in Dataset S1. The biomass reaction was defined as the objective function of the CBM method Flux Balance Analysis (FBA; [29]). FBA looks for a flux distribution *v* that maximizes the objective function (Equation 1) subject to steady-state, thermodynamic and growth medium constraints:
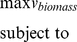
(1)

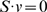
(2)

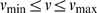
(3)


Equation 2 imposes the steady state constraints on the system, assuming that the metabolite concentrations remain constant in time. Thermodynamic constraints determining the reaction directionalities are accounted for via the flux limits 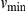 and 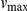 in Equation 3. The uptake and secretion of a pre-defined set of metabolites from and to the environment is facilitated via the definition of exchange reactions in the stoichiometric matrix. The growth medium is defined via an upper bound on the glucose uptake exchange reaction (as the carbon source) and by allowing an unlimited uptake flux of oxygen, sodium, potassium, calcium, iron, chlorine, phosphate, sulfate and ammonia (based on the RPMI- 1640 medium definition; as none of these substances can be used as a carbon source). Growth yield (growth rate divided by the glucose uptake rate), oxygen uptake and lactate secretion rates were computed under a wide range of glucose uptake rates (varying from 0 to 1.55 umol/mgDW/h, the uptake achieving maximal growth rate) using Flux Variability Analysis (FVA) [41], allowing us to determine minimal and maximal flux bounds on the reactions of interest.

### Accounting for enzyme solvent capacity

A constraint on the total enzyme concentration was added to the biomass production FBA model: 
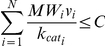



The enzyme mass (per mg dry weight (DW) of cells) required to maintain the flux in the i-th reaction (*v_i_* [mmol/(mgDW*h)]) is given by the product of *v_i_* and the enzyme's molecular weight (*MW_i_* [mg/mmol]) divided by its turnover number (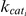 [1/h]) [21]. The limit on the total metabolic enzyme mass (*C* = 0.078 [mg/mgDW]) was estimated based on dry cell weight protein biomass measurements (0.779 [mg/mgDW]; [42]) multiplied by the fraction of metabolic genes out of the total cellular protein mass, which was evaluated as the sum of metabolic gene expression readouts divided by the total sum of gene expression readouts ([33]; equal to 0.1). Notably, the reliability of this value was validated based on a recently published protein abundance dataset ([43], Text S1). In order to account for positive fluxes only, each bidirectional reaction was split into two unidirectional reactions, resulting in a total of 4,894 reactions. Enzyme molecular weights were obtained from the BRENDA database ([44]; Dataset S2) while turnover number data was taken from BRENDA and from the SABIO-RK databases ([45]; Dataset S3), and assigned as following: each reaction with a known Enzyme Commission (EC) number was queried against BRENDA for the maximal human wild-type *k_cat_* value. In case a human *k_cat_* value was not available, the maximal non-human wild-type turnover number was assigned. In case BRENDA data was not available, the SABIO-RK database was used in a similar manner. As a result, 729 reactions were assigned with *k_cat_* values while the other 4,165 reactions were assigned with the median *k_cat_* value across the set of known *k_cat_* values (25 1/s).

### Pathway activity analysis

Flux distributions were computed under maximal growth rates in the three growth phases (phase I – 0.0243 1/h; phase II – 0.0515 1/h; phase III – 0.0557 1/h). For each phase, the median flux distribution across 1000 different uniform samples was calculated using ACHR sampling [46]. Mean pathway flux was calculated as the mean flux across the reactions belonging to the pathway of interest. Data on relative metabolomic measurements for lactate, and on relative transcriptomic measurements for genes which are important for mitochondrial biogenesis (PGC-1-α, NRF-1, TFAM and ATP5E) was taken from [32].

### Correlation with gene expression data

Gene expression readouts for 1,269 metabolic genes across 60 cell lines from the NCI-60 collection [33] were correlated with enzyme concentrations predicted by (i) a stoichiometric only model and by (ii) a model accounting also for enzyme solvent capacity. Given a flux distribution vector *v*, for each reaction *i*, the enzyme concentration 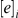 needed to maintain its flux 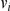 (the i-th entry in *v*) was calculated as the product of
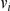
and the molecular weight of the enzyme catalyzing this reaction (denoted 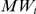), divided by its turnover number (denoted 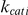), that is, 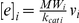. Total enzyme concentrations (per gene) were given by summing the enzyme concentrations across all of the reactions associated with the gene of interest (*i.e.* reactions catalyzed by enzymes encoded by this gene), based on a gene-to-reaction mapping given in the human metabolic model. The Spearman correlation between the gene expression vector and inferred enzyme concentration vector was calculated for the two models in each of the 60 cell lines. The robustness of the results was validated against 1,000 uniformly sampled flux distributions from the solution spaces of the two models using ACHR sampling [46].

### Modeling amino-acid uptakes

Each of the 20 amino acids was added, in turn, to the growth media, resulting in 20 different maximal biomass production rates calculated based on (i) the stoichiometric model, and on (ii) a model additionally accounting for the solvent capacity constraint. The maximal amino-acid uptake rate was set to the same uptake rate as glucose; the results are shown to be robust to the choice of this value (Text S1).

## Supporting Information

Dataset S1 Human biomass composition.
(XLSX)

Dataset S2 Enzyme molecular weight data for the reactions in the model.
(XLSX)

Dataset S3 Enzyme turnover number data for the reactions in the model.
(XLSX)

Text S1 Validating the robustness of the results to various model parameters and exploring additional changes in the model.
(DOC)
