# Supplementary material for: Genome-Scale Metabolic Modeling Elucidates the Role of Proliferative Adaptation in Causing the Warburg Effect
Source: PLoS Comput Biol. 2011 Mar 10;7(3):e1002018. doi: 10.1371/journal.pcbi.1002018 (PMC3053319; doi:10.1371/journal.pcbi.1002018)
Supplement: Text S1 — Validating the robustness of the results to various model parameters and exploring additional changes in the model. (DOC) [file pcbi.1002018.s004.doc]

Text S1

**Genome-scale metabolic modeling elucidates the role of proliferative adaptation in causing the Warburg effect**

Tomer Shlomi^1^*, Tomer Benyamini^2^*, Eyal Gottlieb^3^, Roded Sharan^2^, Eytan Ruppin^2,4^

^1^Computer Science Department - Technion - Israel Institute of Technology, Haifa 32000, Israel

^2^The Blavatnik School of Computer Science – Tel Aviv University, Tel Aviv 69978, Israel

^3^The Beatson Institute for Cancer Research, Glasgow, G61 1BD, UK

^4^The Sackler School of Medicine – Tel Aviv University, Tel Aviv 69978, Israel

*Equal contribution

**Following the work by Vander Heiden *et al.***

We first follow Vander Heiden *et al.* [1] and rigorously examine whether the structure of the entire human metabolic network and the stoichiometry of its reactions directly lead to the Warburg effect due to cellular proliferation, the latter represented by the demands for the production of a single biomass constituent, palmitate. To recapitulate, Vander Heiden *et al.* claimed that the high number of NADPH molecules required to synthesize palmitate (compared to the very few ATP molecules required) would lead to a high uptake of glucose (7 molecules of glucose per one molecule of palmitate), whose complete oxidation would generate ATP in excess in the proliferating cell [1]. Thus, as a counteraction, the cells resort to enhanced glycolysis and lactate secretion (and manifest the Warburg effect).

To reexamine this hypothesis while aiming to avoid potential biases due to the set of analyzed reactions, we utilize a genome-scale human metabolic stoichiometric model that accounts for 3,742 reactions [2]. In difference from Vander Heiden *et al.* who manually inspected only several central pathways, we perform here a large scope analysis of the human model. Maximizing the palmitate production yield using this model (by setting the palmitate production reaction as the FBA objective function) reveals that all glucose carbon atoms can be either incorporated into palmitate or to be completely oxidized to CO_2_ without any need to resort to lactate secretion: specifically, our stoichiometric simulation shows that actually only 4.48 glucose molecules (26.88 carbons) are needed for the production of 1 palmitate molecule (Supp. Figure 1), and that no lactate production is required in the process. Analyzing the flux distribution in the model we find that: (i) the palmitate carbon requirements are satisfied by 4 glucose molecules (24 carbons); (ii) the palmitate NADPH requirements (14 molecules) are satisfied by the malic enzyme (which transforms cytosolic malate to pyruvate; 8.25 NADPH molecules) and by the pentose phosphate pathway (5.75 NADPH molecules coupled with the secretion of 2.87 carbons via CO_2_). Notably, we found that Vander Heiden *et al.* did not account for two additional important enzymes – cytosolic malate dehydrogenase and mitochondrial pyruvate carboxylase (colored in red in Supp. Figure 1) which allow efficient NADPH production via a cycle that utilizes the cytosolic malic enzyme. In addition, there are other efficient NADPH production alternatives that were not accounted for by Vander Heiden *et al.*:(i) through the pentose-phosphate pathway (using G-6-P isomerase in the backward direction; colored in green in Supp. Figure 1Error: Reference source not found) and (ii) using the cytosolic isocitrate dehydrogenase (colored in blue in Supp. Figure 1), while recycling carbons via the malate-aspartate shuttle and the TCA cycle. Both pathways allow high palmitate production yield without requiring lactate to be secreted.

**Robustness of flux predictions to variations in the turnover rates**

In order to account for the solvent capacity constraint, our model requires prior knowledge regarding the turnover rates of all enzymes in the human metabolic network model. Since turnover rates were found for only 729 reactions in the network (extracted from the BRENDA [3] database and from the SABIO-RK database [4]), the remaining 4165 reactions were assigned with the median value of the known turnover rates (in the analysis described in the main text; note that reversible reactions were duplicated to two irreversible ones). To investigate the robustness of the model that accounts for enzyme solvent capacity to the uncertainty in the kinetic parameters we performed 1,000 random samplings of kinetic parameters for the 4165 reactions with unknown turnover rates, and repeated the prediction of Warburg effect characteristics with each sampled parameter set (Supp. Figure 2). The samples were taken from a log-normal distribution whose mean and standard deviation were determined from the distribution of known turnover rates for the remaining 729 reactions [5]. The resulting metabolic predictions show similar trends to the ones obtained with the median turnover rate, as presented in the main text.

**Estimating the enzyme mass limit based on proteomics**

As mentioned in the Methods (main text), we estimated the upper bound on the total enzyme mass (C = 0.078 [mg/mgDW]) based on dry cell weight protein biomass measurements (0.779 [mg/mgDW]) multiplied by the fraction of metabolic genes out of the total cellular protein mass, which was evaluated as the sum of metabolic gene expression readouts divided by the total sum of mRNA gene expression readouts (equal to 0.1). In order to validate the reliability of the estimated value of 0.1 – the metabolic enzyme fraction out of the total cellular proteome, we used a recently published protein abundance data for 1,051 proteins, out of which 94 are metabolic enzymes, which is considered to be the most comprehensive one up to date [6]. This dataset doesn’t obviously cover the entire human proteome, but treating it as an unbiased sample for a first approximation, the mass fraction of the metabolic enzymes out of the total protein mass gives 0.0881. Notably, the mRNA expression fraction (measured in the same work) gave 0.0883. Altogether, these findings are leading to the conclusion that 0.1 value is not a bad approximation.

**Adding glutamine as an additional carbon source to glucose**

Glutamine is frequently used as a carbon source, secondary to glucose, by cancerous tumors [7]. Therefore, we re-examined our results in a model accounting for both glucose and glutamine as carbon sources. We demonstrate that our original results remain robust to this modification: We observe a qualitatively similar three-phase behavior including a moderate yield decrease in the medium growth rates (Supp. Figure 3A, phase II) accompanied by an increased oxygen uptake (Supp. Figure 3B, phase II), followed by a sharp yield decrease in the higher growth rates along with lactate secretion and a sharp decrease in the oxygen uptake (Supp. Figure 3, phase III). Notably though, the picture here is slightly more complex, as there are two “peaks” in the oxygen uptake plot during phase II. As in the case of the glucose-only model, correlation values with gene expression data of the NCI-60 cell lines also show robustness to the addition of glutamine to the growth medium: the model accounting for enzyme solvent capacity has a mean correlation of 0.281, while the model accounting for stoichiometry only had a mean correlation of only 0.104.

**Studying the individual growth contribution of all amino acids**

Maximal biomass production rates were calculated by including each of the 20 amino acids in the growth medium (one by one) in addition to glucose as a main potential source of carbon. This inclusion was implemented and studied across a variety of bounds on amino-acid uptake rates, both in (i) a strictly stoichiometric model, where the different uptake limits have no effect on the order of growth rates obtained with the different amino-acids (Supp. Figure 4A) and by (ii) a model accounting for the solvent capacity constraint, where the order of the amino-acids changes between the lower and the higher uptake rates, until reaching the solvent capacity limit (where increasing the uptake limit does not affect the maximal growth rate; Supp. Figure 4B). Still, glutamine provides the highest growth rates of all amino acids across all uptake rates in our model.

In order to validate the robustness of this result to the choice of enzyme turnover numbers, we sampled 1000 turnover number distributions for the set reactions for which this parameter is unknown, out of the set of known turnover numbers, similarly to what was done in the “Flux Robustness” section above. As observed in Supp. Figure 5, our original finding holds and glutamine achieves the highest mean fraction of growth rate improvement obtained vs. the glucose growth rate (mean improvement fraction of 0.66).

**Accounting for cell maintenance ATP production**

Accounting for a constant (growth rate independent) production rate of ATP for cell maintenance purposes is important when accounting for the cellular energetic potential, and cellular metabolism in general [8]. In fact, the study of the Warburg effect by [9] was previously criticized by [8] for not accounting for ATP maintenance requirements. We therefore investigated the robustness of our model by accounting for a constant ATP maintenance requirement (via an ATP hydrolysis reaction constrained to have a minimal flux of 1.0625 mmol/gDW/h [10]). A non-constant growth yield is observed even when using the stoichiometric model alone – since a constant amount of carbon uptake is needed to satisfy the maintenance ATP production, independently of the growth rate (the yield is calculated as the growth rate divided by the carbon uptake rate; Supp. Figure 6A). Nevertheless, using the model that accounts also for enzyme solvent capacity, we witness a behavior very similar to that obtained in the original model, where maintenance ATP were not accounted for: a decrease of the yield at the higher growth rates accompanied by an increased oxygen uptake (corresponding to Phase II, Supp. Figures 6,7), followed by a shaper decrease, accompanied by lactate secretion and decreased oxygen uptake (Phase III, Supp. Figures 6,7). Furthermore, repeating the correlation with NCI-60 expression data analysis, the significant advantage of the model that accounts for solvent capacity over the stoichiometric-only one is retained (stoichiometric model: mean correlation = 0.101, mean p-value = 5.47e-4; model with enzyme solvent capacity: mean correlation = 0.281, mean p-value = 8.69e-22; Wilcoxon test p-value = 3.56e-21).

**Accounting for a limit on the mitochondrial solvent capacity**

In our work we accounted for a solvent capacity constraint assuming there’s a limited space for proteins in the entire cell. However, especially in eukaryotic organisms, many proteins are confined to certain compartments (e.g. mitochondria). To examine how this additional limitation may influence the results, we added a mitochondrial solvent capacity constraint:

$$\sum_{i\in R_{M}} \frac{MW_{i}v_{i}}{k_{cat_{i}}}\leq M$$

where *R_M_* is the set of mitochondrial reactions (defined as reactions involving at least one mitochondrial metabolite), and *M =0.048* is an upper bound on the mitochondrial mass fraction in the cell. *M* was estimated as the product of (i) the total number of mitochondrial in per gram dry weight of a rat liver cell (3e11; [11]) and (ii) the mitochondrial dry mass (13.6e-14; [12]). Re-running the original analyses showed that the new constraint had no effect on the results – i.e. it is less constraining than the original solvent capacity constraint on the entire cell.

**Accounting for enzyme volumes instead of enzyme masses**

Our original solvent capacity constraint accounts for enzyme mass fractions required to maintain a given flux, bounded by a bound on the total enzyme mass fraction. However, as mentioned in the main text, our formulation is based on a work by Beg *et al.* [13] where enzyme volumes were accounted for instead of enzyme masses. In order to account for enzyme volumes, Beg *et al.* linked between the mass of each enzyme to the volume it occupies, using a *specific volume* constant. In their work, Beg *et al.* use an identical specific volume for all of the enzymes in the *E. coli* metabolic network. However, in the recent work by Vazquez *et al.* [9], they assume different specific volumes for the mitochondrial and the glycolytic enzymes, which may affect the result. We therefore reformulated the solvent capacity constraint as following:

$$S_{M}\sum_{i\in R_{M}} \frac{MW_{i}v_{i}}{k_{cat_{i}}}+S_{C}\sum_{i\notin R_{M}} \frac{MW_{i}v_{i}}{k_{cat_{i}}}\leq V$$

where *R_M_* is the set of mitochondrial reactions (defined as reactions involving at least one mitochondrial metabolite), *S_M_* = 2.9 mL/g and *S_C_* = 0.79 mL/g are the specific volumes of the mitochondria and the cytoplasm, respectively (obtained from [9]); *V* = 0.1059 mL/gDW is a bound on the total cell volume (per gram dry cell mass) available for metabolic enzymes, obtained as the volume of a single HeLa cell (2.425e-9 mL [14]) divided by its dry weight (2.29e-9 g; [15]) and multiplied by the fraction of metabolic enzymes out of the entire cell mass evaluated as the sum of metabolic gene expression readouts divided by the total sum of gene expression readouts ([16]; equal to 0.1). Re-running the simulations showed qualitatively similar results (Supp. Figure 8).

**References**

1. Vander Heiden MG, Cantley LC, Thompson CB (2009) Understanding the Warburg Effect: The Metabolic Requirements of Cell Proliferation. Science 324: 1029-1033.

2. Duarte NC, Becker SA, Jamshidi N, Thiele I, Mo ML, et al. (2007) Global reconstruction of the human metabolic network based on genomic and bibliomic data. Proc Natl Acad Sci USA 104: 1777 - 1782.

3. Schomburg I, Chang A, Ebeling C, Gremse M, Heldt C, et al. (2004) BRENDA, the enzyme database: updates and major new developments. Nucl Acids Res 32: D431-433.

4. Rojas I, Golebiewski M, Kania R, Krebs O, Mir S, et al. (2007) Storing and annotating of kinetic data. In Silico Biol 7: S37-44.

5. Vazquez A, Beg QK, Demenezes MA, Ernst J, Bar-Joseph Z, et al. (2008) Impact of the solvent capacity constraint on E. coli metabolism. BMC Syst Biol 2: 7.

6. Vogel C, de Sousa Abreu R, Ko D, Le S-Y, Shapiro BA, et al. (2010) Sequence signatures and mRNA concentration can explain two-thirds of protein abundance variation in a human cell line. Mol Syst Biol 6.

7. DeBerardinis RJ, Mancuso A, Daikhin E, Nissim I, Yudkoff M, et al. (2007) Beyond aerobic glycolysis: transformed cells can engage in glutamine metabolism that exceeds the requirement for protein and nucleotide synthesis. Proc Natl Acad Sci U S A 104: 19345-19350.

8. Locasale JW, Cantley LC (2010) Altered metabolism in cancer. BMC Biol 8: 88.

9. Vazquez A, Liu J, Zhou Y, Oltvai Z (2010) Catabolic efficiency of aerobic glycolysis: The Warburg effect revisited. BMC Syst Biol 4: 58.

10. Kilburn DG, Lilly MD, Webb FC (1969) The energetics of mammalian cell growth. J Cell Sci 4: 645-654.

11. Allard C, Mathieu R, De Lamirande G, Cantero A (1952) Mitochondrial population in mammalian cells. I. Description of a counting technic and preliminary results on rat liver in different physiological and pathological conditions. Cancer Res 12: 407-412.

12. Glas U, Bahr GF (1966) Quantitative study of mitochondria in rat liver. Dry mass, wet mass, volume, and concentration of solids. J Cell Biol 29: 507-523.

13. Beg QK, Vazquez A, Ernst J, de Menezes MA, Bar-Joseph Z, et al. (2007) Intracellular crowding defines the mode and sequence of substrate uptake by Escherichia coli and constrains its metabolic activity. Proceedings of the National Academy of Sciences 104: 12663-12668.

14. Zhao L, Kroenke CD, Song J, Piwnica-Worms D, Ackerman JJH, et al. (2008) Intracellular water-specific MR of microbead-adherent cells: the HeLa cell intracellular water exchange lifetime. NMR in Biomedicine 21: 159-164.

15. Park K, Jang J, Irimia D, Sturgis J, Lee J, et al. (2008) 'Living cantilever arrays' for characterization of mass of single live cells in fluids. Lab on a Chip 8: 1034-1041.

16. Lee JK, Havaleshko DM, Cho H, Weinstein JN, Kaldjian EP, et al. (2007) A strategy for predicting the chemosensitivity of human cancers and its application to drug discovery. Proc Natl Acad Sci U S A 104: 13086-13091.

Supp. Figure 1: The flux distribution achieving maximal palmitate production yield. The two enzymes not accounted for in the Vander Heiden *et al.* analysis appear here in red. Dashed arrows represent zero flux. Two other alternatives for NADPH production appear in green and in blue.

Supp. Figure 2: Predicted maximal growth yield (A; per unit of glucose uptake), Oxygen uptake (B) and Lactate secretion (C) under different growth rates based on the strictly stoichiometric model (red line), solvent-capacity constrained model using the median *k_cat_* (blue line), and the solvent capacity constrained model using randomly sampled turnover rates (green line). The green lines display the mean across the 1,000 samples for each growth rate; the error bars represent the standard deviation of the mean.

Supp. Figure 3: Predicted growth yield (green lines, A) and O2 uptake (blue lines, B) and lactate secretion rates (red lines, B) across different growth rates. Dashed lines represent predictions generated using stoichiometry only, while solid lines stand for predictions of the solvent-capacity constrained model.

Supp. Figure 4: Maximal amino acid uptake rates vs. maximal growth rates predicted by (A) the stoichiometric model and (B) the solvent capacity constrained model. Each line corresponds to a different amino acid; In B, the glutamine line is above the other lines at any uptake rate value.

Supp. Figure 5: The increase in proliferation rate achievable by the increased uptake of each of the 20 amino-acids in addition to glucose (relative to the baseline growth rate achieved when only glucose is available), as predicted by the stoichiometric model (A), by the model accounting for the solvent capacity constraint using the median turnover number (B) and by the solvent capacity constrained model using random turnover number samples (C; bar length represents the mean value across the 1000 samples). Glutamine uptake (highlighted in yellow) enables to achieve the highest increase in growth rate according to the solvent capacity model (B and C).

Supp. Figure 6: Predicted growth yield (A, green lines), uptake/secretion fluxes (B: green – glucose uptake, blue – O2 uptake, red – lactate secretion), and zooming-in on oxygen uptake flux (C) across different growth rates in the ATP-maintenance version of the model. Dashed lines represent predictions generated using stoichiometry only, while solid lines stand for predictions of the solvent capacity constrained model. The results demonstrate an increased oxygen uptake (at growth rate = 0.052, accompanied by a yield decrease comparing to the stoichiometric model) followed by lactate secretion (growth rates 0.052 – 0.057, accompanied by an even sharper yield decrease).

Supp. Figure 7: Pathway activity differences as predicted by the model accounting for the solvent capacity constraint and for maintenance-associated ATP production. Results were generated as described in the methods section with the following growth rates: phase I – 0.001 1/h, phase II - 0.051 1/h, phase III – 0.0569 1/h.

Supp. Figure 8: Predicted growth yield (green lines, A) and O2 uptake (blue lines, B) and lactate secretion rates (red lines, B) across different growth rates (flux normalized by the glucose uptake rate). Dashed lines represent predictions generated using stoichiometry only, while solid lines stand for predictions of the solvent-capacity constrained model accounting for volumes (instead of mass concentrations).
